# Associations Between Key Psychosocial Stressors and Viral Suppression and Retention in Care Among Youth with HIV in Rural South Africa

**DOI:** 10.1007/s10461-021-03198-9

**Published:** 2021-02-24

**Authors:** Lindsey M. Filiatreau, Audrey Pettifor, Jessie K. Edwards, Nkosinathi Masilela, Rhian Twine, F. Xavier Gómez-Olivé, Nicole Haberland, Chodziwadziwa Whiteson Kabudula, Sheri A. Lippman, Kathleen Kahn

**Affiliations:** 1 Department of Epidemiology, Gillings School of Global Public Health, University of North Carolina at Chapel Hill, 170 Rosenau Hall, CB #7400, 135 Dauer Drive, Chapel Hill, NC 27599-7400 USA; 2 MRC/Wits Rural Public Health and Health Transitions Research Unit (Agincourt), School of Public Health, Faculty of Health Sciences, University of the Witwatersrand, Johannesburg, South Africa; 3 Population Council, New York, NY USA; 4 Division of Prevention Science, Department of Medicine, University of California, San Francisco, San Francisco, CA USA

**Keywords:** Youth with HIV, Viral suppression, Retention in care, Adherence, Psychosocial health, Mental health

## Abstract

Despite improvements in access to antiretroviral treatment over the past decade, sub-optimal HIV care outcomes persist among youth with HIV (YWH) in rural South Africa. Psychosocial stressors could impede improved HIV treatment outcomes within this population. We linked self-reported psychosocial health and demographic data from a cross-sectional survey conducted among YWH aged 12–24 in rural South Africa to individual medical record data, including facility visit history and viral load measurements. Poisson regression with robust standard errors was used to estimate the associations between five psychosocial stressors- heightened depressive symptoms (Center for Epidemiological Studies-Depression scale scores ≥ 16), lower social support (Medical Outcomes Social Support Scale scores ≤ 38), lower resilience (Conner-Davidson Resilience Scale scores ≤ 73), lower self-esteem (Rosenberg Self-Esteem Scale scores ≤ 21), and higher perceived stress (Sheldon Cohen Perceived Stress Scale scores ≥ 10)- and viral non-suppression (viral load ≥ 400 copies/mL) and loss to care (no documented clinic visits within the 90 days prior to survey), separately. A total of 359 YWH were included in this analysis. The median age of study participants was 21 (interquartile range: 16–23), and most were female (70.2%), single (82.4%), and attending school (54.7%). Over a quarter of participants (28.1%) had heightened depressive symptoms. Just 16.2% of all participants (n = 58) were lost to care at the time of survey, while 32.4% (n = 73) of the 225 participants with viral load data were non-suppressed. The prevalence of non-suppression in individuals with lower self-esteem was 1.71 (95% confidence interval: 1.12, 2.61) times the prevalence of non-suppression in those with higher self-esteem after adjustment. No meaningful association was observed between heightened depressive symptoms, lower social support, lower resilience, and higher perceived stress and viral non-suppression or loss to care in adjusted analyses. Retention in care and viral suppression among YWH in rural South Africa are below global targets. Interventions aimed at improving viral suppression among YWH should incorporate modules to improve participant’s self-esteem as low self-esteem is associated with viral non-suppression in this setting. Future studies should longitudinally explore the joint effects of co-occurring psychosocial stressors on HIV care outcomes in YWH and assess meaningful differences in these effects by age, gender, and route of transmission.

**Supplementary Information:**

The online version contains supplementary material available at 10.1007/s10461-021-03198-9.

## Introduction

Young people continue to bear a disproportionate burden of the HIV epidemic worldwide. In South Africa specifically, over one-third of all new HIV infections occur among youth aged 15 to 24 [1–3]. The most recent country estimates suggest that only 39.9% of youth with HIV (YWH) in this age range are on antiretroviral treatment (ART) [1, 3]. Despite improvements in ART accessibility over the past decade and adoption of the World Health Organization’s Universal Test and Treat recommendation in September 2016 [4], poor HIV care outcomes persist among South African YWH [5].

Adolescence and young adulthood are periods of physical and mental maturation, as well as identity experimentation [6]. These stages in the life course are often characterized by poor mental health outcomes, limited social support, and increased vulnerability to stigma and discrimination [6–9]. A growing body of research suggests that young peoples’ psychosocial well-being could directly affect HIV care outcomes (e.g., retention in care and viral suppression) [9–13]. In addition, the World Health Organization now recommends integrated, comprehensive mental health services for all people with HIV, and calls for peer support programs for YWH, specifically [14, 15]. However, studies estimating the association between psychosocial stressors and HIV care outcomes and interventions aimed at improving HIV care outcomes through psychosocial interventions have shown mixed effects among YWH [12, 16–21]. For example, 98.6% of YWH were retained on treatment after implementation of a peer counseling and psychosocial support program at healthcare facilities and schools in Kenya, as compared to just 54.4% pre-implementation [21]. However, there were no differences in the timing of linkage to care among YWH pre- and post-implementation of the program [21].

In many low-and middle-income countries, particularly in rural settings, improving HIV care outcomes in YWH is further complicated by limited access to both mental healthcare and age-appropriate clinical services [22–24]. Young people often fear stigmatization by healthcare providers and their peers, have difficulty accessing services outside school hours, or are deterred from seeking sexual health services by parental consent policies [25, 26].

If we are to meet the Joint United Nations Program on HIV and AIDS’ 95–95–95 targets, it is essential to identify specific psychosocial stressors that impede improved HIV care outcomes among YWH in the era of Universal Test and Treat. In doing so, psychosocial interventions to improve HIV treatment outcomes in YWH can be tailored to meet the specific mental health needs of YWH. This study aims to address existing gaps in the literature through a comprehensive exploration of potential psychosocial correlates of sub-optimal HIV treatment outcomes in South African YWH. More specifically, we estimate the association between five psychosocial stressors- heightened depressive symptoms, low social support, low self-esteem, low resilience, and high perceived stress- and loss to care and viral non-suppression in YWH in rural South Africa.

## Methods

### Setting

This study was conducted in the Agincourt Health and Socio-Demographic Surveillance System study area (HDSS) located in rural Mpumalanga Province, South Africa. The study area is approximately 500 km northeast of Johannesburg [27] and home to approximately 120,000 individuals [28]. The population is characterized by high levels of poverty [29], intimate partner violence [30], and HIV infection [31]. Access to public sector services and economic opportunities post-schooling is limited, contributing to high rates of work-related migration particularly in youth exiting the school system [32].

Nine publicly funded healthcare facilities provide medical services to a majority of study area residents. In these facilities, access to primary healthcare and ART is free of charge. However, patient wait times often exceed national standards and there is limited to no differentiated care for young people [33]. Psychological services, including access to mental health medications, are seriously limited and restricted to district hospitals some 30–40 km away. Several social workers provide services within the study area. However, the scope of their work and the extensive populations they serve make mental healthcare extremely difficult to access for a majority of residents [27].

### Study Population

Young people aged 12–24 who had a documented HIV positive test result in one of the nine HDSS public healthcare facilities were recruited for study participation. Recruitment was based on HIV test results recorded in Tier.net, the national electronic HIV/ART monitoring platform [34, 35], or the HDSS-Clinic Link System used to track patient outcomes, described below. To maximize study participation among individuals both in and out of HIV care, recruitment was conducted using a three-pronged approach: through trained study staff, area medical providers, and home-based care providers who regularly interact with YWH in their coverage areas. Individuals were ineligible to participate if they received most of their HIV care outside the nine publicly funded health facilities, or were pregnant, participating in any other HIV care studies, or outside the ages of 12 to 24 at the time of the survey. Eligibility was determined through participant self-report.

### Data Collection

#### Youth Survey

Our team conducted a cross-sectional survey between April and August 2019 among YWH meeting the inclusion criteria. Participant surveys were translated and back-translated from English to Xitsonga, the local language, and administered by locally hired research assistants fluent in both languages. Surveys were administered in the participant’s language of choice and captured data on individuals’ experiences with the healthcare system, living with HIV (self-reported HIV care outcomes, HIV-related stigma), psychosocial stressors (depressive symptoms, social support, self-esteem, resilience, and perceived stress), experiences of violence, and substance use. Participant responses were recorded electronically on study tablets by research assistants.

#### HDSS-Clinic Link

Survey data were linked to participants’ medical records captured in the HDSS-Clinic Link System that has been previously described in detail [36, 37]. Briefly, the HDSS-Clinic Link is a population-based clinical care database that covers consenting/assenting patients seeking HIV-specific services or chronic care in all nine publicly funded health facilities within the study area. Data typists, stationed at each of the facilities since 2014, consent/assent patients seeking care on a daily basis. After obtaining written informed consent/assent, clinical visit data and patient demographic data is captured in the Clinic Link System and linked to HDSS census data in real-time. Data typists continually update clinical records data as individuals return for services. All data are de-identified before the provision of analytic datasets.

### Exposures

All exposures of interest were ascertained on the youth survey. *Depressive symptoms* were measured using the 20-item Center for Epidemiological Studies-Depression (CES-D) scale [38], which has been used among South African adolescents with high validity and reliability (α = 0.90) [39]. Cronbach’s alpha in the current study was acceptable at 0.76. Possible scores range from 0 to 60 and were dichotomized at 16 to represent those with or without (reference) heightened depressive symptoms [40]. We also explored dichotomizing scores at the median (see Supplementary Content).

*Social support* was captured using a modified 8-item Medical Outcomes Social Support Survey [41]. The expanded version of this scale has been widely used in South Africa [42–45] with high reliability among YWH specifically (α = 0.85) [43]. Cronbach’s alpha in the current study was high at 0.92. Possible scores range from 8 to 40 with higher scores indicative of greater social support. Scores were dichotomized at the median to represent those with higher (reference) versus lower social support, given there is no standard scale cut point. We also dichotomized scores at the 25th percentile to explore extremely low social support as a psychosocial stressor (see Supplementary Content).

*Self-esteem* was captured using the 10-item Rosenburg Self-Esteem Scale [46], which has been previously used throughout South Africa with moderate to high reliability (α ranging from 0.78 to 0.94) [47–50]. Cronbach’s alpha in the current study was acceptable at 0.78. Possible scores range from 0 to 30 with higher scores indicative of greater self-esteem [46]. Scores were dichotomized at the median to represent those with higher (reference) versus lower self-esteem, given there is no standard scale cut point. We also dichotomized scores at the 25th percentile to explore extremely low self-esteem as a psychosocial stressor (see Supplementary Content).

*Resilience* was captured using the 25-item Conner Davidson Resilience scale [51], which has been previously used among South African adolescents with high reliability (α = 0.93) [52]. Cronbach’s alpha in the current study was high at 0.94. Possible scores range from 0 to 100 with higher scores indicative of greater resilience. Scores were dichotomized at the median to represent those with higher (reference) versus lower resilience, given there is no standard scale cut point. We also dichotomized scores at the 25th percentile to explore extremely low resilience as a psychosocial stressor (see Supplementary Content).

*Perceived stress* was captured using the 10-item Sheldon Cohen Perceived Stress scale [53]. Versions of this scale have been previously used among South African adolescents with moderate reliability (α = 0.74) [54, 55]. Cronbach’s alpha in the current study was 0.75. Possible scores range from 0 to 40 with higher scores indicative of greater perceived stress [53, 56]. Scores were dichotomized at the median to represent those with higher versus lower (reference) perceived stress, given there is no standard scale cut point. We also dichotomized scores at the 75th percentile to explore extremely high perceived stress as a psychosocial stressor (see Supplementary Content).

### Outcomes

*Loss to care* was ascertained through participants’ medical records, captured in the HDSS-Clinic Link System. Participants with no documented clinic visits in the 90 days prior to survey participation were considered out of care (i.e., “lost to care”). This definition is consistent with a lapse in medication coverage defined in the South Africa national HIV adherence guidelines [57].

*Viral non-suppression* was ascertained using viral load measurements recorded in the HDSS-Clinic Link System. Measurements of 400 copies/mL and above were considered virally non-suppressed [58, 59]. We included viral load measurements taken within the 90 days prior to study interview or subsequent to study interview but before the outcome ascertainment closure date in March 2020. Measurements from a wide range of dates were included because viral load testing is recommended just once per year in South Africa and often taken inconsistently. Outcomes were considered missing for participants with no recorded viral load within this time frame. When a participant had multiple measurements within the described time frame the last reported viral load measurement was utilized.

### Covariates

Adjusted analyses controlled for age, gender, and time since diagnosis (years) at the time of the survey, which were identified a priori [10, 12, 60]. Age and gender were captured through self-report and time since diagnosis was calculated using the time between the first documented date of diagnosis in the HDSS-Clinic Link System and the survey date. Age and time since diagnosis were operationalized according to functional form assessment using visual inspection and comparison of model fit. Age was modeled on the log-linear scale in loss to care models and categorically in viral non-suppression models. Time since diagnosis was modeled quadratically in loss to care models and on the log-linear scale in viral non-suppression models.

### Ethical Review and Informed Consent

Participants aged 18 and over were required to provide written informed consent to be eligible for study participation. Those under the age of 18 were required to provide written assent, and have written informed consent from a parent or guardian at least 18 years of age. Ethical approval was obtained from the University of North Carolina at Chapel Hill’s Institutional Review Board, the University of the Witwatersrand’s Human Research Ethics Committee, and the Mpumalanga Provincial Health Research Committee. Clinic and community access were facilitated through the HDSS Public Engagement Office.

### Analysis

Descriptive statistics (counts/proportions or medians/interquartile ranges [IQRs]) were used to describe the study population. Comparisons of covariate distribution by gender and each HIV care outcome were conducted using Pearson’s chi-squared tests for categorical variables and Wilcoxon rank-sum tests for continuous measures.

To account for missingness of individual scale items on each psychosocial scale, single imputation was conducted using the mean of the individual’s non-missing scale items, given no more than 10% of the total scale items were missing [61]. For individuals with a missing date of HIV diagnosis, date of birth was used in calculating time since diagnosis for those under the age of 15 and those self-reporting perinatal infection. For those self-reporting horizontal transmission, and those aged 15 or older with an unknown route of transmission, the first documented HIV clinic visit date was used to calculate time since diagnosis.

Multiple imputation was used to account for missingness of viral suppression status (n = 134, 37.3%), under the assumption that data were missing at random. We used Pearson’s chi-squared tests to identify measured covariates associated with viral non-suppression in the complete case analysis and measured covariates associated with missingness. One hundred imputations were conducted using the monotone logistic method for each exposure of interest. Each imputation model included the exposure of interest, covariates in the final analytic model (age, gender, and time since diagnosis), and two predictors of viral non-suppression (one viral load measurement from the year preceding the study period, and a second from the year before that). Sensitivity analyses in which all missing values were assumed to be either non-suppressed or suppressed were also conducted (Supplementary Table 1).

Poisson regression with robust variance estimators was used to estimate the unadjusted and adjusted associations between each of the five psychosocial stressors (heightened depressive symptoms, low social support, low self-esteem, low resilience, and high perceived stress) and each outcome of interest (loss to care and viral non-suppression), separately. When estimating the associations between each psychosocial stressor and viral non-suppression, estimates obtained from the imputed datasets were combined and summarized using standard multiple imputation techniques. These estimates were compared to estimates obtained from the complete case analysis and results from each sensitivity analysis (Supplementary Table 1). The original dataset was utilized in estimating the associations between each psychosocial measure and loss to care. All analyses were conducted using SAS version 9.4 (Cary, North Carolina).

## Results

A total of 362 individuals participated in the study survey. Three participants were excluded from the analytic dataset due to incomplete questionnaires. Among the 359 included participants, 70.2% were female and the median age was 21 (IQR: 16–23). Most participants were single (82.4%) and attending school (54.7%). Among those not enrolled in school (n = 158), most were unemployed (93.0%) (Table 1). Female participants were older, more likely to have completed secondary school, more likely to be in a relationship, and less likely to be in school than male participants (data not shown).

**Table 1:** Socio-demographic characteristics of 359 youth with HIV in rural Mpumalanga Province, South Africa stratified by HIV care outcomes

	Suppression status*		p-value	Retention in care status		p-value	Total (n = 359) n (%)
	Non-suppressed (n = 73) n (%)	Suppressed (n = 152) n (%)		Lost to care (n = 58) n (%)	In care (n = 301) n (%)		
Age (median/IQR)	20 (16–22)	21 (17–23)	0.04	22 (20–23)	20 (16–23)	< 0.00	21 (16–23)
< 15	10 (13.7)	25 (16.4)	< 0.01	0 (0.0)	53 (17.6)	< 0.01	53 (14.8)
15–19	25 (34.2)	24 (15.8)		14 (24.1)	77 (25.6)		91 (25.3)
20–24	38 (52.1)	103 (67.8)		44 (75.9)	171 (56.8)		215 (59.9)
Gender							
Male	26 (35.6)	46 (30.3)	0.42	10 (17.2)	97 (32.2)	0.02	107 (29.8)
Female	47 (64.4)	106 (69.7)		48 (82.8)	204 (67.8)		252 (70.2)
Education							
None/some primary	20 (27.4)	33 (21.7)	0.03	3 (5.2)	74 (24.6)	< 0.01	77 (21.5)
Completed primary	37 (50.7)	58 (38.2)		29 (50.0)	133 (44.2)		162 (45.1)
Completed secondary	16 (21.9)	61 (40.1)		26 (44.8)	94 (31.2)		120 (33.4)
Orphanhood status							
Non-orphan	38 (52.0)	69 (45.4)	0.07	29 (50.0)	135 (44.9)	0.51	164 (45.7)
Single orphan	18 (24.7)	60 (39.5)		17 (29.3)	112 (37.2)		129 (35.9)
Double orphan	17 (23.3)	23 (15.1)		12 (20.7)	54 (17.9)		66 (18.4)
Marital status*							
Single	61 (84.7)	122 (80.3)	0.42	43 (74.1)	252 (84.0)	0.07	295 (82.4)
Partnered	11 (15.3)	30 (19.7)		15 (25.9)	48 (16.0)		63 (17.6)
Employment*							
Working for pay	2 (2.9)	7 (4.7)	< 0.05	0 (0.0)	11 (3.7)	< 0.01	11 (3.2)
Current student	46 (65.7)	72 (48.0)		21 (38.2)	170 (57.8)		191 (54.7)
Unemployed	22 (31.4)	71 (47.3)		34 (61.8)	113 (38.4)		147 (42.1)

*IQR* interquartile range

*Missing: suppression status = 134; marital status = 1; employment = 10

Over a quarter of study participants (28.1%) had heightened depressive symptoms (Table 2). The median social support score was 38 (IQR: 32–40), with 144 individuals (40.1%) scoring the maximum score of 40, representing social support all or most of the time (Table 2). The median resilience score was 73 (IQR: 64–80), slightly below a score of 75 which is representative of individuals feeling resilient “fairly often” (Table 2). The median self-esteem score was 21 (IQR: 18–24), slightly above a score of 20 which is representative of individuals who “agree” to each positive statement about their self-worth (Table 2). The median perceived stress score was 10 (IQR: 6–15) which is representative of individuals “almost never” feeling stressed (Table 2). There were no meaningful differences in scores by gender (data not shown).

**Table 2:** Psychosocial characteristics of 359 young people living with HIV in rural Mpumalanga Province, South Africa stratified by HIV care outcomes

	Suppression status*		p-value	Retention in care status		p-value	Total (n = 359) n (%)
	Non-suppressed (n = 73) n (%)	Suppressed (n = 152) n (%)		Lost to care (n = 58) n (%)	In care (n = 301) n (%)		
Depression (median/IQR)	13 (12–15)	13.5 (12–16)	0.65	12 (10–15)	13 (12–16)	0.10	13 (12–16)
Depressed	18 (24.7)	51 (33.6)	0.18	14 (24.1)	87 (28.9)	0.46	101 (28.1)
Non-depressed	55 (75.3)	101 (66.4)		44 (75.9)	214 (71.1)		258 (71.9)
Social support (median/IQR)	38 (33–40)	38 (32–40)	0.19	35.7 (29–40)	38 (32–40)	0.06	38 (32–40)
Lower support	37 (50.7)	83 (54.6)	0.58	35 (60.3)	152 (50.5)	0.17	187 (52.1)
Higher support	36 (49.3)	69 (45.4)		23 (39.7)	149 (49.5)		172 (47.9)
Resilience (median/IQR)	72 (63–69)	72 (64–79)	0.76	75 (63–83)	72 (65–80)	0.49	73 (64–80)
Lower resilience	41 (56.2)	86 (56.6)	0.95	26 (44.8)	161 (53.5)	0.23	187 (52.1)
Higher resilience	32 (43.8)	66 (43.4)		32 (55.2)	140 (46.5)		172 (47.9)
Self-Esteem (median/IQR)	20 (18–23)	21 (18–24)	0.05	20 (17–24)	21 (18–24)	0.33	21 (18–24)
Lower self-esteem	50 (68.5)	80 (52.6)	0.02	35 (60.3)	170 (56.5)	0.59	205 (57.1)
Higher self-esteem	23 (31.5)	72 (47.4)		23 (39.7)	131 (43.5)		154 (42.9)
Perceived Stress (median/IQR)	10 (5–15)	10 (6–16.5)	0.93	11 (8–16)	9 (5–14)	< 0.05	10 (6–15)
Higher perceived stress	39 (53.4)	79 (52.0)	0.83	38 (65.5)	148 (49.2)	0.02	186 (51.8)
Lower perceived stress	34 (46.6)	73 (48.0)		20 (34.5)	153 (50.8)		173 (48.2)

*IQR* interquartile range

*Missing: suppression status = 134

30 study participants with a recorded positive HIV test result self-reported they were HIV negative (8.4%). Nearly half of participants with self-reported data on mode of transmission (n = 151; 46.9%) said they did not know how they became infected, and 105 (32.6%) self-reported perinatal infection. The median age at diagnosis was 17 (IQR: 9–20), and median time since diagnosis was 3 years (IQR: 1–7) (Table 3). Time since diagnosis was longer in males when compared to females and males were more likely to report perinatal transmission (data not shown).

**Table 3:** Characteristics of HIV infection of 359 young people living with HIV in rural Mpumalanga Province, South Africa stratified by HIV care outcomes

	Suppression status*		p-value	Retention in care status		p-value	Total (n = 359) n (%)
	Non-suppressed (n = 73) n (%)	Suppressed (n = 152) n (%)		Lost to care (n = 58) n (%)	In care (n = 301) n (%)		
Self-reported mode of infection*							
Perinatal	27 (39.7)	41 (28.9)	0.22	13 (28.9)	92 (33.2)	0.83	105 (32.6)
Heterosexual	8 (11.8)	32 (22.5)		10 (22.2)	52 (18.8)		62 (19.3)
Other	1 (1.5)	2 (1.4)		1 (2.2)	3 (1.1)		4 (1.2)
Don’t know	32 (47.0)	67 (47.2)		21 (46.7)	130 (46.9)		151 (46.9)
Years since diagnosis (median/IQR)	5 (3–8)	3 (1–6)	< 0.00	3 (1–6)	4 (2–7)	0.24	3 (1–7)

*IQR* interquartile range

*Missing: suppression status = 134; self-reported mode of transmission = 37

58 individuals (16.2%) were lost to care, and 73 of 225 individuals (32.4%) with viral load measurements were virally non-suppressed. 78 of the 301 participants (25.9%) who were engaged in care at the time of survey had a missing viral load measurement. While female participants were more likely to be lost to care than male participants, no meaningful difference was observed in viral non-suppression by gender (Table 1).

**Fig. 1:**
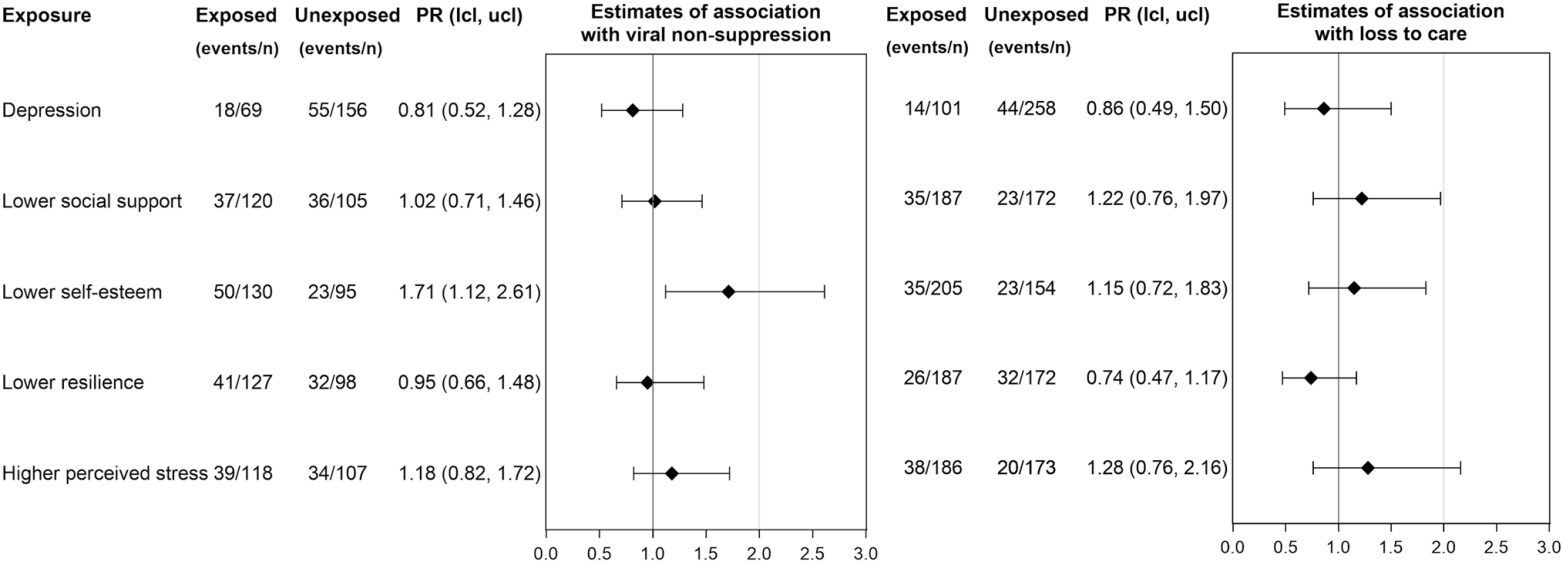
Adjusted estimates of association between key psychosocial stressors and viral non-suppression and loss to HIV care in 359 youth with HIV in rural Mpumalanga Province, South Africa. All estimates are adjusted for age, gender, and time since diagnosis (years). *PR* prevalence ratio; *lcl* lower 95% confidence interval limit; *ucl* upper 95% confidence interval limit

The prevalence of viral non-suppression in individuals with lower self-esteem was 1.71 (95% confidence interval (CI): 1.12, 2.61) times the prevalence in individuals with higher self-esteem after adjustment (Fig. 1). No meaningful association was observed between heightened depressive symptoms, lower social support, lower resilience, or higher perceived stress and viral non-suppression. Results from the complete case analysis and sensitivity analyses trended in the same direction as results from multiply imputed analyses (Supplementary Table 1).

In crude analyses, the prevalence of loss to care among participants with higher perceived stress was 1.77 (95% CI: 1.07, 2.91) times the prevalence of loss to care among those with lower perceived stress (Supplementary Table 2). However, this association was attenuated in adjusted analyses at 1.28 (95% CI: 0.76, 2.16) (Fig. 1). No meaningful association was observed between heightened depressive symptoms, lower social support, lower resilience, or lower self-esteem and loss to care (Fig. 1). Results from the sensitivity analyses trended in the same direction as the results in Fig. 1.

## Discussion

This study is one of the first to comprehensively explore psychosocial measures associated with loss to care and viral non-suppression in YWH in the era of Universal Test and Treat. Findings highlight the fact that retention in care and viral suppression remain a challenge among YWH in rural South Africa, with non-suppression of particular concern. Nearly 85% of study participants had at least one clinic visit within the 90 days prior to study participation. However, 37.3% had no documented viral load measurement within the study period. Among those with a viral load measurement over 30% were non-suppressed. These estimates remain well below the 95–95–95 target, indicating a clear need for the identification of factors impeding retention in care and viral suppression among YWH.

We hypothesized that five psychosocial measures would be associated with loss to care and viral non-suppression among YWH in this context. However, of the five psychosocial measures explored, low self-esteem was the only measure associated with either outcome in adjusted analyses. Self-esteem was relatively high among study participants when compared to adolescents with HIV in Kenya and Uganda [62]. Still, the prevalence of viral non-suppression among individuals with lower self-esteem was 1.71 times the prevalence of non-suppression in those with higher self-esteem when controlling for relevant covariates. On the other hand, depressive symptoms were relatively common within the population when compared to other YWH in South Africa [7], yet no meaningful association was observed between heightened depressive symptoms and viral non-suppression or loss to care in the study population. Perceived stress was lower, and resilience and social support were higher relative to similar populations [63], potentially accounting for the lack of association between these measures and viral non-suppression or loss to care in adjusted analyses.

We believe these complex results could be attributable to a number of factors. First, evidence suggests the association between psychosocial stressors and HIV treatment outcomes may vary by gender, age, or route of HIV transmission [64–70]. For example, in a study of 113 adults with HIV in the United States, not belonging to an HIV support group was significantly associated with ART non-adherence among men but not among women [64]. A qualitative study among 30 YWH found unique psychosocial barriers to treatment adherence among those perinatally versus behaviorally infected [66]. While we controlled for gender, age, and time since diagnosis, which can serve as a proxy for mode of transmission in YWH, we were unable to assess sub-group associations (i.e., effect modification) due to concerns about positivity and small sample sizes. It is possible significant associations between psychosocial stressors and HIV treatment outcomes that occurred in specific sub-groups were obscured in the population overall. Second, psychosocial stressors are often entangled, co-occurring, and can interact to exacerbate sub-optimal treatment outcomes beyond what is expected when adding the effects of each exposure on the outcome [71–73]. It is possible self-esteem was driving other sub-optimal psychosocial outcomes within the population, thereby confounding the relationship between the other measures and outcomes of interest. However, we were unable to explore the temporal relationship and potential interactive effects between measured psychosocial stressors due to the cross-sectional nature of the youth survey and concerns about small sample sizes and lack of positivity. Again, this could have obscured other meaningful associations between the explored psychosocial stressors and care outcomes of interest.

Overall, our findings contribute to a growing body of literature that suggests some psychosocial stressors are associated with HIV care outcomes in YWH. However, caution should be taken in drawing conclusions about the effects of psychosocial well-being on HIV care outcomes more broadly. The significance of the relationship between specific stressors and treatment outcomes vary by setting and population of interest. For example, the odds of viral suppression in a study among YWH in Malawi did not differ by depression status [19]. However, lower social support decreased odds of suppression in the population (OR: 0.95, 95% CI: 0.93, 0.99) [19]. In a study among Rwandan YWH, no association was observed between self-reported depressive symptoms, mixed anxiety and depression, or self-esteem and treatment non-adherence. However, caregiver report of depressive symptoms was associated with non-adherence (OR: 1.02; 95% CI: 1.01, 1.04) [12].

This study has several limitations that should be considered. Study outcomes were ascertained using medical record data and are subject to data quality issues common to medical record databases (e.g., missingness). While some missingness may be attributable to lapses in data capturing, medical record audits suggest viral load measurements are taken inconsistently within this context. It is also plausible that missingness is associated with unmeasured factors that directly influence viral suppression. However, we believe the inconsistencies in clinic-based viral load measurement likely account for the bulk of the missingness observed in suppression data. To minimize the amount of missing viral load measurements, we included measurements taken within the 3 months prior to study interview. This may raise temporality concerns because some viral load measurements preceded exposure ascertainment. Loss to care was also captured at the time of exposure ascertainment. However, psychosocial measures are generally stable over short periods (e.g., 3 months) [74, 75] and our over-arching goal was to identify meaningful associations between psychosocial stressors and HIV care outcomes as opposed to drawing causal conclusions about the explored relationships. This study precludes YWH who did not have a documented positive test result in Tier.net or the HDSS-Clinic Link System. While our three-pronged approach to participant recruitment allowed us to trace individuals who had fallen out of care, it is probable that our team located fewer individuals who were lost to care, and that individuals who were lost were less likely to participate. As a result, we may have missed the most vulnerable individuals in our study population, potentially contributing to the high prevalence of psychosocial wellness observed in the population.

Future studies that aim to identify psychosocial factors associated with loss to care or viral non-suppression in YWH should be adequately powered to assess effect modification by gender, age, and mode of transmission. Further, longitudinal studies that enroll and follow YWH from the point of diagnosis, or entry into care in the case of those perinatally infected, could allow for causal estimation of the effects of each psychosocial stressor, or co-occurring psychosocial stressors, on specific HIV care outcomes over time, particularly during the time of transition around adolescence.

## Conclusions

Retention in care and viral suppression among YWH in rural Mpumalanga Province, South Africa are below global targets. Improving young people’s self-esteem could increase adherence to daily treatment regimens, thereby improving viral suppression. However, the relationship between psychosocial well-being and specific HIV care outcomes is complex. Future studies should aim to longitudinally explore and identify causal effects of key psychosocial stressors, relative to other, potentially more impactful factors, on specific HIV care outcomes by age, gender, and mode of transmission.

## Supplementary Information

Below is the link to the electronic supplementary material.

Supplementary file1 (DOCX 17 KB)
