# Supplementary material for: Associations Between Key Psychosocial Stressors and Viral Suppression and Retention in Care Among Youth with HIV in Rural South Africa
Source: AIDS Behav. 2021 Feb 24;25(8):2358–68. doi: 10.1007/s10461-021-03198-9 (PMC8222008; doi:10.1007/s10461-021-03198-9)
Supplement: Supplementary file 1 — Supplementary file1 (DOCX 17 KB) [file 10461_2021_3198_MOESM1_ESM.docx]

**Supplementary Table 1. Estimates of association between psychosocial measures and viral non-suppression in 359 youth living with HIV in rural Mpumalanga Province, South Africa**

| **Exposure (cut point)** | **Complete case**  **uPR** | **Complete case**  **aPR^*^** | **Imputation**  **aPR^*^** | **Sensitivity analysis 1: missing non-suppressed**  **aPR^*^** | **Sensitivity analysis 2: missing suppressed**  **aPR^*^** |
| --- | --- | --- | --- | --- | --- |
| Depression (16) | 0.74 (0.47, 1.16) | 0.76 (0.48, 1.19) | 0.81 (0.52, 1.28) | 0.83 (0.66, 1.03) | 0.87 (0.53, 1.42) |
| Depression (median) | 0.92 (0.63, 1.34) | 0.97 (0.67, 1.41) | 1.03 (0.70, 1.51) | 0.89 (0.75, 1.06) | 1.15 (0.75, 1.75) |
| Lower social support (median) | 0.90 (0.62, 1.31) | 0.91 (0.64, 1.30) | 1.02 (0.71, 1.46) | 0.95 (0.80, 1.14) | 0.99 (0.66, 1.49) |
| Lower social support (25%ile) | 0.80 (0.51, 1.26) | 0.92 (0.58, 1.45) | 0.98 (0.64, 1.49) | 1.01 (0.82, 1.23) | 0.82 (0.50, 1.34) |
| Lower self-esteem (median) | 1.59 (1.05, 2.41) | 1.50 (1.00, 2.25) | 1.71 (1.12, 2.61) | 1.14 (0.96, 1.37) | 1.64 (1.05, 2.56) |
| Lower self-esteem (25%ile) | 1.28 (0.87, 1.89) | 1.17 (0.81, 1.71) | 1.26 (0.86, 1.85) | 1.12 (0.93, 1.34) | 1.23 (0.81, 1.88) |
| Lower resilience (median) | 0.99 (0.68, 1.44) | 1.00 (0.69, 1.44) | 0.95 (0.66, 1.36) | 0.87 (0.73, 1.03) | 1.15 (0.76, 1.73) |
| Lower resilience (25%ile) | 1.06 (0.70, 1.62) | 1.07 (0.71, 1.61) | 0.99 (0.66, 1.48) | 0.97 (0.79, 1.18) | 1.05 (0.66, 1.66) |
| Higher perceived stress (median) | 1.04 (0.71, 1.52) | 1.13 (0.78, 1.64) | 1.18 (0.82, 1.72) | 1.01 (0.84, 1.20) | 1.11 (0.73, 1.73) |
| Higher perceived stress (75%ile) | 0.95 (0.61, 1.46) | 1.02 (0.66, 1.56) | 1.07 (0.71, 1.62) | 1.00 (0.81, 1.22) | 1.03 (0.64, 1.66) |

*adjusted for age (categorical), sex, time since diagnosis (log-linear)

uPR: unadjusted prevalence ratio; aPR: adjusted prevalence ratio

**Supplementary Table 2. Estimates of association between psychosocial measures and loss**

**to care in 359 youth living with HIV in rural Mpumalanga Province, South Africa**

| **Exposure (cut point)** | **uPR** | **aPR^*^** |
| --- | --- | --- |
| Depression (16) | 0.81 (0.47, 1.42) | 0.86 (0.49, 1.50) |
| Depression (median) | 0.63 (0.39, 1.01) | 0.66 (0.41, 1.06) |
| Lower social support (median) | 1.40 (0.86, 2.27) | 1.22 (0.76, 1.97) |
| Lower social support (25%ile) | 1.63 (1.02, 2.62) | 1.38 (0.84, 2.26) |
| Lower self-esteem (median) | 1.14 (0.71, 1.85) | 1.15 (0.72, 1.83) |
| Lower self-esteem (25%ile) | 1.47 (0.91, 2.36) | 1.30 (0.83, 2.05) |
| Lower resilience (median) | 0.75 (0.47, 1.20) | 0.74 (0.47, 1.17) |
| Lower resilience (25%ile) | 1.20 (0.72, 2.01) | 1.09 (0.64, 1.83) |
| Higher perceived stress (median) | 1.77 (1.07, 2.91) | 1.28 (0.76, 2.16) |
| Higher perceived stress (75%ile) | 1.67 (1.04, 2.70) | 1.34 (0.83, 2.18) |

*adjusted for age (log-linear), sex, time since diagnosis (quadratic)

uPR: unadjusted prevalence ratio; aPR: adjusted prevalence ratio
